# MSC Pretreatment for Improved Transplantation Viability Results in Improved Ventricular Function in Infarcted Hearts

**DOI:** 10.3390/ijms23020694

**Published:** 2022-01-08

**Authors:** Mark F. Pittenger, Saman Eghtesad, Pablo G. Sanchez, Xiaoyan Liu, Zhongjun Wu, Ling Chen, Bartley P. Griffith

**Affiliations:** 1 Department of Surgery, School of Medicine, University of Maryland, Baltimore, MD 21201, USA; saman14313@yahoo.com (S.E.); sanchezpg@upmc.edu (P.G.S.); liu9832129@gmail.com (X.L.); zwu@som.umaryland.edu (Z.W.); 2 Department of Biochemistry, School of Medicine, University of Maryland, Baltimore, MD 21201, USA; 3 Department of Cardiothoracic Surgery, University of Pittsburgh Medical Center, Pittsburgh, PA 15260, USA; 4 Departments of Physiology and Medicine, School of Medicine, University of Maryland, Baltimore, MD 21201, USA; lchen685@gmail.com

**Keywords:** MSC, pretreatment, prosurvival factors, myocardial infarct, improved ejection fraction, rat model, mesenchymal stem cells, mesenchymal stromal cells

## Abstract

Many clinical studies utilizing MSCs (mesenchymal stem cells, mesenchymal stromal cells, or multipotential stromal cells) are underway in multiple clinical settings; however, the ideal approach to prepare these cells in vitro and to deliver them to injury sites in vivo with maximal effectiveness remains a challenge. Here, pretreating MSCs with agents that block the apoptotic pathways were compared with untreated MSCs. The treatment effects were evaluated in the myocardial infarct setting following direct injection, and physiological parameters were examined at 4 weeks post-infarct in a rat permanent ligation model. The prosurvival treated MSCs were detected in the hearts in greater abundance at 1 week and 4 weeks than the untreated MSCs. The untreated MSCs improved ejection fraction in infarcted hearts from 61% to 77% and the prosurvival treated MSCs further improved ejection fraction to 83% of normal. The untreated MSCs improved fractional shortening in the infarcted heart from 52% to 68%, and the prosurvival treated MSCs further improved fractional shortening to 77% of normal. Further improvements in survival of the MSC dose seems possible. Thus, pretreating MSCs for improved in vivo survival has implications for MSC-based cardiac therapies and in other indications where improved cell survival may improve effectiveness.

## 1. Introduction

While it was once thought that the heart had no ability to repair or regenerate, studies have provided evidence of cardiomyocyte replacement in the normal heart as well as during post-injury remodeling that involve new cells migrating into the heart or provided from the cardiac tissue itself [1,2,3], and mouse cardiomyocytes are known to experience significant proliferation during adolescence [4,5]. However, in the damaged heart, the endogenous repair mechanisms are generally not adequate to repair or replace the amount of tissue lost during infarction, particularly as aging progresses. Since the first study to engraft progenitor cells in the infarcted myocardium was reported in 1992 [6], many investigators have grafted exogenous cells into the infarcted heart, attempting to replace the damaged cells, to augment function, and to prevent deleterious remodeling. Current strategies for cellular heart repair include identifying appropriate cell type(s); efficient delivery methods; local tissue conditions receptive to the cellular therapy (peri-infarct); improved engraftment; graft survival; and ultimately, clinical translation and appropriate patient selection. A variety of stem/progenitor cells are under study that may have the appropriate characteristics to augment or replace cardiac cells lost following infarction. Such potential replacement cells include mesenchymal stem cells (MSCs) [7,8,9,10,11,12], c-kit+ cardiac progenitor cells [13,14,15], as well as embryonic stem cells (ESCs) [16,17,18,19,20] and induced pluripotent stem cells (iPSCs) [21,22,23,24,25,26,27]. There are currently more than 583 clinical studies grafting cells into the heart, 129 utilizing MSCs of which 77 are completed, active or recruiting [28,29], and http://clinicaltrials.gov (accessed on 1 December 2021). Poor survival of the stem/progenitor cells transferred into the impaired heart tissue has been one of the obstacles to successful cell therapy for the injured myocardium. The in vivo infarcted tissue is dramatically different from the in vitro culture environment used for cell expansion [30,31]. The implanted/infused exogenous cells encounter “new” conditions in vivo including major changes in their biochemical microenvironment, nutrients, and oxygen levels that can cause cell stress [32,33]. In an infarcted heart, the oxygen concentration level at the infarct site is near ~1–2%, while in the nearby normally perfused healthy myocardium, it is closer to 13% [34]. The consequences of this infarct hypoxia are free radical accumulation, subsequent hydrogen peroxide production, membrane lipid peroxidation, inflammation, cardiomyocyte death, and damaged cardiac tissue. Therefore, the fragile cellular drug must be “hardened” to withstand changes in their immediate environment when moved from culture conditions to the damaged heart [35,36,37,38,39]. Here, we have tested an anti-apoptosis strategy to provide a survival advantage to MSCs introduced into the cardiac injury site.

MSCs (mesenchymal stem cells, multipotential stromal cells, or mesenchymal stromal cells) are multipotent cells commonly obtained from the adult bone marrow or adipose tissue that are capable of multilineage differentiation including adipocytes, osteocytes, chondrocytes, and endothelial and muscle cells in vitro [7,40,41,42] and that can produce important growth factors and cytokines. When MSCs are implanted into damaged cardiac tissue of experimental models of myocardial infarction, the treated hearts have improved cardiac remodeling, vascularity, and ejection fraction and greater preservation of myocardium at risk [43,44]. Mechanistically, the MSCs provide strong anti-inflammatory and immunomodulatory effects through the release of multiple paracrine factors that reduce the deleterious effects of tissue injury on the nearby surviving cardiac tissue, and clinical studies have provided evidence that MSCs can improve the outcomes of myocardial infarction patients, although the reported clinical effects have been modest [45,46,47,48]. However, in these reported clinical studies, many aspects of the cellular therapy had not been optimized. In particular, the survival of in vitro cultured MSCs is quite limited in the in vivo environment following delivery into cardiac tissue, and we believe that improving MSC engraftment should increase their long-term therapeutic potential. Some studies have used gene-modified MSCs overexpressing Bcl-2, GATA-4, or Akt in cardiac injury models [49,50,51]. Several studies have reported the effects of certain anti-apoptotic and pro-survival factors (PSFs) with different cell types in animal models of cell therapy [52,53] and using anti-apoptotic pretreatment had a positive effect on the survival of hESCs following myocardium injection [44]. These studies defined that there are several apoptotic pathways and that, if one pertinent pathway was blocked, cell death occurred by another pathway such that it was necessary to block all apoptotic pathways to increase cell survival.

The present study seeks to improve immediate engraftment and graft survival of MSCs. Biochemicals and growth factors that have been used in patients previously were used to prevent MSC apoptosis and included insulin-like growth factor 1 (IGF-1), immunoregulatory agent cyclosporine A, anti-apoptotic agents Bcl-Xl BH4 and pinacidil, and a caspase-3 inhibitor ZVAD. A heat shock protocol 24 h prior to in vivo delivery is also known to improve transplanted cell survival. We tested the outcomes of in vitro experiments of rat MSCs (rMSCs) pretreated with these factors to enhance their survival compared to untreated rMSCs and analyzed the effects of untreated compared with treated MSCs in vivo in the rat permanent ligation infarct model.

## 2. Results

### 2.1. Differentiation of rMSCs of GFP Rats

**Figure 1:**
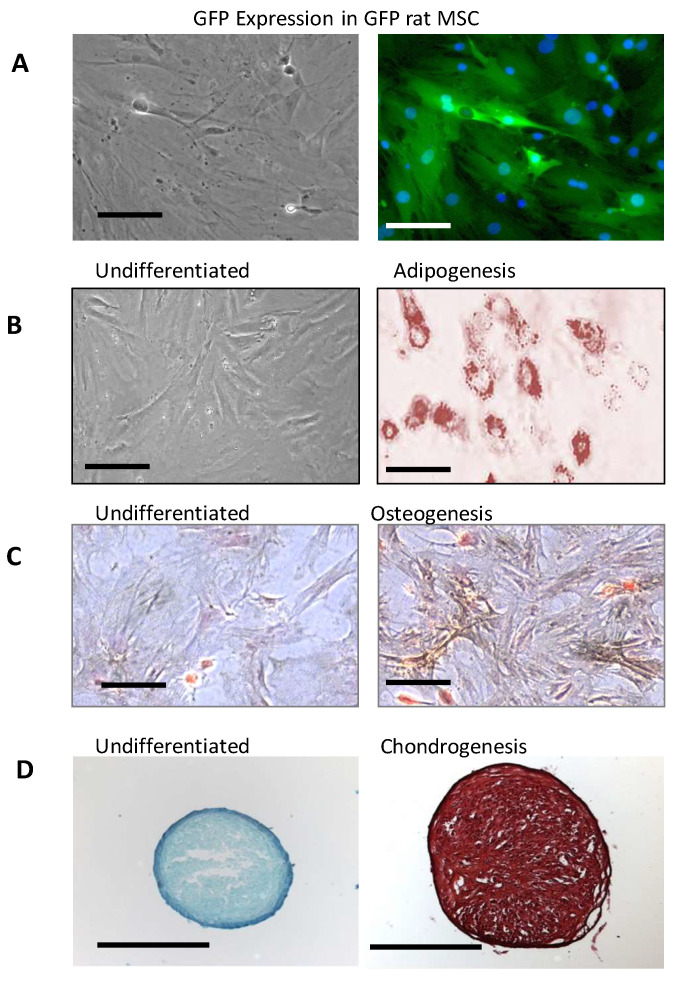
Characterization of GFP-rat mesenchymal stem cells (rMSC) in vitro. (**A**) Expression of green fluorescent protein (GFP) in rMSC from bone marrow of Lewis GFP+ transgenic rats in culture. (**B**–**D**) Differentiation of GFP rat MSCs under adipogenic conditions demonstrated by Oil-Red-O staining (**B**) and osteogenic conditions after staining calcium deposition (dark particles) by the method of von Kossa (**C**), and chondrogenic differentiation in pellet culture format after sectioning and staining with safranin O (**D**). Multilineage differentiation was tested on three different occasions by two different researchers. The GFP expression continues in the differentiated MSCs, but its visualization is not seen well in the stained samples. Panels A, B, and C, Bar = 100 µm. Panel D, Bar = 1 mm.

The GFP-rMSCs used in these studies (Figure 1) were characterized by flow cytometry and for their ability to differentiate into adipocytes, osteoblasts, and chondrocytes. These newly isolated GFP expressing rMSCs have not been studied previously so it was important to demonstrate their multilineage differentiation. The GFP-MSCs successfully underwent adipogenesis in 14 days. Lipid vesicles were evident at 7 days and increased in number and size by 14 days (Figure 1B is day 14). For osteogenic differentiation, the GFP-rMSCs were examined at 14 days for alkaline phosphatase and von Kossa staining and histochemistry showed increased alkaline phosphatase activity and von Kossa silver deposition. For chondrogenic differentiation, the cells expressed extensive extracellular matrix that stained with safranin O. The GFP-rMSCs continue to express GFP after lineage differentiation (not shown).

### 2.2. Challenge with Hydrogen Peroxide Leads to Apoptotic MSC Death

**Figure 2:**
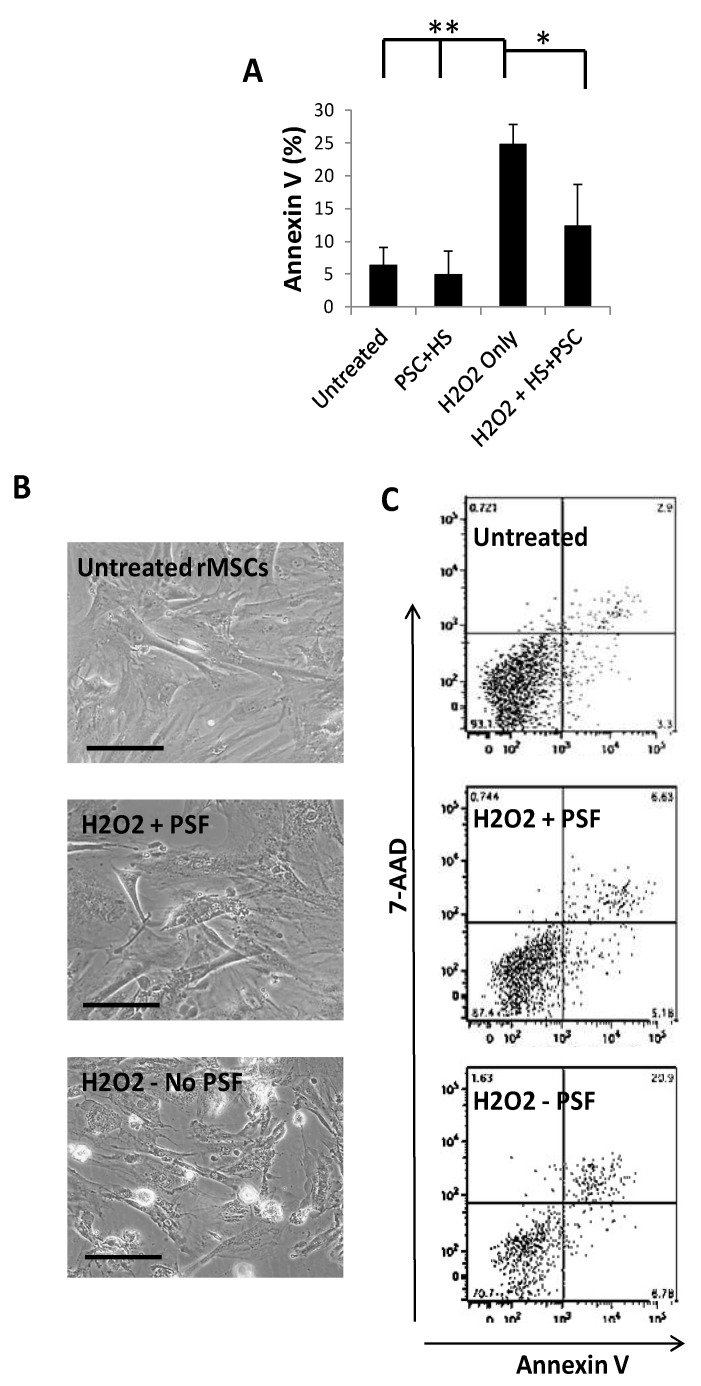
Annexin V staining analysis of apoptosis with in vitro stress assays. (**A**) Compiled results of GFP-rMSC apoptosis at 1 h following addition of hydrogen peroxide to culture medium. (**B**) Representative images of GFP-rMSCs apoptosis for in vitro stress assays. (**C**) Representative flow cytometry results of staining for annexin V. Untreated-2.9% apoptotic, PSF + H_2_O_2_ treated-5.6% apoptotic, H_2_O_2_ treated-20.9% apoptotic. Samples were in triplicate and performed on two occasions. ** indicates *p* < 0.5 H_2_O_2_ treated compared with untreated or PSF, * indicates *p* < 0.5 H_2_O_2_ treated compared with PSF treated MSCs. Bar =100 µm.

To test the effect of hydrogen peroxide challenge on the GFP-rMSCs, the cells were cultured until they were about 70–80% confluent and hydrogen peroxide-containing medium was prepared by serial dilutions to find the effective concentration for the subsequent studies based on dose–response experiments. The effects of hydrogen peroxide on GFP-rMSCs were examined by testing for apoptosis with the annexin V detection and 7-AAD labeling of the cells at 2 h. A concentration of 0.85 mM hydrogen peroxide for 2 h resulted in approximately 50% cell death of the GFP-rMSCs. Therefore, samples were analyzed at 1 h of treatment to facilitate handling. As shown in Figure 2A–C, when compared with the untreated cells, HS/PSF-treated GFP-rMSCs showed a significant 2–3-fold decrease in 7-ADD staining when exposed to hydrogen peroxide.

### 2.3. Protein Expression Differences in Hydrogen Peroxide Challenged GFP-rMSCs

**Figure 3:**
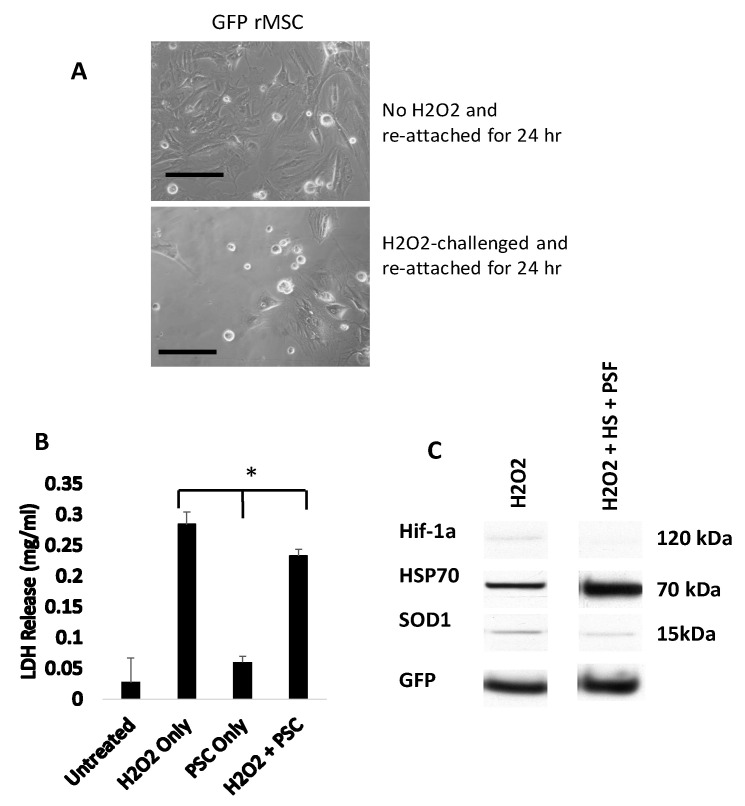
GFP-rMSCs following in vitro stress assays. (**A**) Trypsinized and re-plated GFP-rMSCs at 24 h following hydrogen peroxide treatment. PSF-treated cells were much more abundant and had begun dividing, but GFP-rMSCs not treated with PSF appeared to have lasting negative effects of H_2_O_2_ exposure. (**B**) Lactate dehydrogenase released into medium of the hydrogen peroxide-treated cells was a means to measure cell death. (**C**) Changes in expression of stress-related proteins Hif-1a, HSP70, and SOD1 in GFP-rMSCs following hydrogen peroxide treatment. * Indicates *p* < 0.5. Experiments were repeated twice. Bar =100 µm.

An apoptotic protein array was used to identify hydrogen peroxide-induced protein expression changes in GFP-rMSC cultures (data not shown). Several pro-apoptotic and stress-related proteins including heat shock protein 70 (HSP70), hypoxia inducible factor-1 alpha (Hif-1α), and superoxide dismutase 1 (SOD1) were expressed at higher levels in hydrogen peroxide-treated cells and, therefore, were further investigated. As indicated in Figure 3C, the expressions of both Hif-1α and SOD1 were decreased when the cells were heat shocked and treated with the pro-survival factors prior to hydrogen peroxide treatment. The expression of HSP70, however, was increased >5 fold in the heat shock + prosurvival treatment group, confirming abundant HSP expression at 24 h. Most of the hydrogen peroxide treated GFP-rMSCs did not survive following typical subculturing with trypsin/EDTA (Figure 3A,B) indicating latent damage.

### 2.4. Prior Treatment with Heat Shock and PSF Improves In Vivo Survival of GFP-rMSCs in the Infarcted Heart

**Figure 4:**
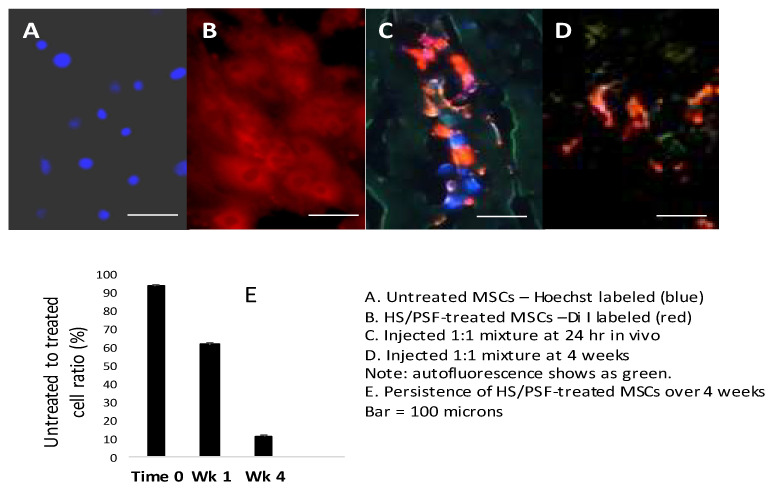
In vivo analysis of cell survival following HS/PSF treatments. (**A**) Hoechst 33,342 labeled untreated GFP-rMSCs and HS/PSF-treated, and Di I labeled GFP-rMSCs in the cell cultures prior to injections into rat hearts. (**B**) Mixed untreated (Hoechst) and HS/PSF-treated (Di I) cells injected into infarcted rat heart and sectioned at 24 h. At this time, equal numbers of untreated and HS/PSF-treated cells could be found. (**C**) However, at 1 week, there were 0.6 Hoechst untreated cells for every HS/PSF-treated cells, and untreated rMSCs declined thereafter. (**D**) At 4 weeks, both groups declined but many more HS/PSF-treated MSCs were present, but very few untreated MSCs were found. At 8 weeks, it was difficult to find any DAPI labeled untreated MSCs, while the HS/PSF-treated MSCs could be found but in reduced numbers compared with the 4-week time point. (**E**) Ratio of HS/PSF-treated GFP-rMSC over untreated GFP-rMSC in rat infarcted heart tissue at 1 and 4 weeks post injections. Bar = 100 µm.

To examine the persistence of HS/PSF-treated versus untreated GFP-rMSCs in the same infarcted animals, a 1:1 mixture of red surface stained DiI labeled HS/PSF-treated and blue nuclear Hoechst 33,342 stained untreated GFP-rMSCs—a total of 2 million cells—was injected. The HS/PSF treated-GFP-rMSCs, which were stained with Di I (red fluorochrome), were found at all time points examined, but both groups diminished with time (Figure 4). The presence of untreated GFP-rMSCs (Hoechst stained) diminished rapidly, and at 1 week, they were only 60% of the number of HS/PSF-treated cells. These untreated cells were difficult to find after 1 week. As shown in Figure 4C, cells from both groups diminished but the untreated to HS/PSF-treated cells decreased more rapidly, indicating an in vivo survival advantage for the HS/PSF-treated GFP-MSCs.

### 2.5. Transplantation of HS/PSF-Treated GFP-rMSC Improved Post-Infarct LV Function

**Figure 5:**
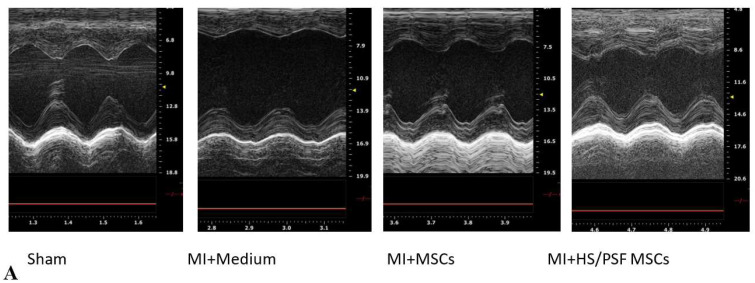

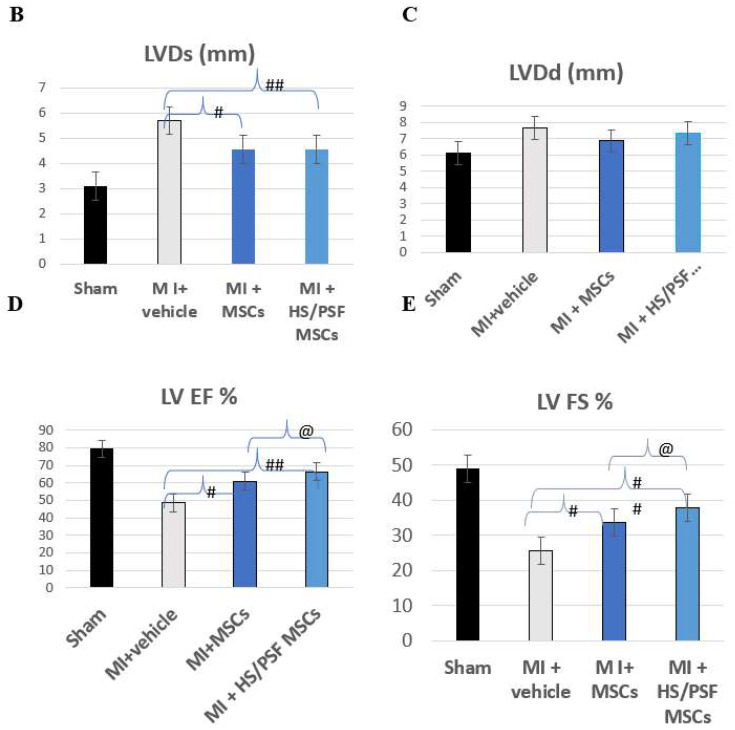
Echocardiography effects of untreated and HS/PSF-treated MSCs in infarct post-infarct hearts. (**A**) Representative M-mode images of four groups. Compared with Sham, MI heart showed significant LV dilatation and reduced wall motion, which was mitigated in MSC-treated hearts; (**B**–**E**) Compared with Sham, MI hearts showed significant increases in diastolic and systolic LV dimension (LVDd and LVDs), indicating LV dilatation, and significant decreases in LV ejection fraction (LVEF) and fractional shortening (LVFS), indicating reduced LV contractility. The LV dilatation and reduced contractility were mitigated in the two MSC treated MI groups compared with MI with the vehicle (medium) treatment. Importantly, compared with untreated MSCs, HS/PSF treated MSCs had improved LVEF and LVFS. #, ## *p* < 0.05 or 0.01 vs. MI medium; and @ *p* < 0.05 compared with MI untreated MSCs. The number of animals in each group was 7 or 8, as given in Table 1. # *p* < 0.05 for MI + vehicle vs. MI + MSCs. ## *p* < 0.01 for MI + vehicle vs. MI + HS/PSF MSCs. @ *p* < 0.05 for MI + MSCs vs. MI + HS/PSF MSCs.

For rat transplantation studies, 32 animals were randomized into 4 groups of 8. However, 3 animals died, each in a different group, so 7 animals were analyzed in the sham, infarct only, and infarct + MSC groups and 8 animals were analyzed in the infarct + treated MSC group. The surgeries were performed over ~8 weeks, usually planned as control, sham, MSC, and treated MSC during each surgery session. Echocardiography was performed at 4 weeks following the surgery, and the data are summarized in Table 1. Compared with the sham surgery with vehicle injection, the ligated rats receiving vehicle injection showed LV dilation as indicated by increases in LVDs (Figure 5D) and LVDd (Figure 5E), and LV hypertrophy as indicated by increases in LVPWd, LV mass, and LV mass index (i.e., LV mass normalized with body weight). LV contractile function deteriorated in the permanently ligated rats with vehicle injection, indicated by decreases in LVEF (Figure 5B) and LVFS (Figure 5C) compared with the sham-operated rats. Myocardial transplantation of either untreated or HS/PSF-treated GFP-rMSCs improved LV contractile function when compared with vehicle alone, as shown by the greater LVEF (Figure 5B), LVFS (Figure 5C), and LVDs (Table 1). However, compared with sham-operated rats, at the delivered dose in this permanent ligation model, the two groups of rats with GFP-rMSC transplantation still showed signs of LV dilation (LVDd—Figure 5E), hypertrophy (LV mass—Table 2), and contractile dysfunction (LVEF, Figure 5B, and LVFS, Figure 5C), which suggests that MSCs partially but not completely prevent the LV changes induced by permanent LAD ligation. In comparisons between the two ligated heart groups receiving MSC transplantation, the MSCs treated with HS/PSF showed significantly greater LVEF (8%) and LVFS (11%), although the differences in other indices were not significant (Table 2). Additionally, as shown in Table 1, the ligated hearts with the HS/PSF-treated GF-MSCs had greater cardiac indices (i.e., SV/BW and CO/BW), compared with the vehicle-injected LAD-ligated hearts. The differences in these cardiac indices between the LAD-ligated hearts with vehicle compared with untreated MSCs were insignificant. Therefore, these results demonstrated that transplantation of GFP-rMSCs with or without HS/PSF pretreatment is beneficial in the preservation of LV contractile function at least to 4 weeks following the LAD ligation, with a greater cardio protection by the HS/PSF treated GFP-rMSCs compared with the untreated GFP-rMSCs.

## 3. Discussion

After myocardial infarction, grams of heart tissue containing billions of cells are lost. Self-repair mechanisms or current therapeutic approaches do not allow for efficient re-cellularization and regeneration of the original structure and cardiac function. However, treatment with exogenous progenitor cells has consistently provided evidence of increased vascularity, preservation of cardiomyocytes, and overall improved LV remodeling. The promising improvements in cardiac pump functions such as ejection fraction have been relatively modest to date, and additional insight is needed to understand the potential/limitations of exogenous cell grafting. Mechanistically, from previous cardiac injury studies as well as work in other fields, MSCs are known to produce factors that reduce inflammation (including TGFβ, PGE2, IDO, TSG6, and IL-1RA) and improve angiogenesis (including VEGF, Ang-1, and FGF2) in damaged tissue and cause a positive effect on recovery and tissue healing [2,8,46,49]. Clinically, despite the many trials utilizing MSCs, there are still fundamental aspects of MSC cellular therapy that need to be addressed and improving the survival of the implanted cells is essential.

In the present study, we sought to improve the immediate survival of transplanted MSCs. The treatment of heat shock combined with a mixture of compounds that inhibit apoptosis were tested. From previous work with hESC transplantation, these compounds plus brief heat shock effectively inhibit cell apoptosis. These compounds together block each of the known apoptotic pathways but using only one or two allowed the alternate pathways to cell death, limiting cell engraftment [44]. The heat shock/prosurvival factor treatment used in this study were tested in vitro with the GFP-rMSCs and the cells benefited from HS/PSF treatment. The modulated expression of SOD and Hif-1α in the HS/PSF-treated GFP-rMSCs speaks to the importance of oxygenation of the target tissue for receiving the in vitro cultured cells normally grown in atmospheric O_2_. This agrees with published work using other types of stem cells and different pre-treatments [49,50,51,52,53]. We used a challenging permanent ligation injury model, and clinical approaches would certainly begin with opening any blocked arteries to improve tissue oxygenation. In the current in vivo study, GFP-rMSCs were delivered by direct injection into the ischemic cardiac tissue rather than systemic delivery. As such, the effects of HS/PSF cell treatment on cardiac engraftment were assessed and not cellular migration through the body or homing to the injury site (although these are important questions.) We observed a substantially increased persistence of the HS/PSF-treated MSCs in the rat hearts compared with the untreated MSCs when a 1:1 mixture of the cells was injected in the same hearts (Figure 4), indicating early cell apoptosis is common in untreated MSCs.

By echocardiographic analysis, the data indicated an overall improvement in the LV contractile function following MSC injection into the infarcted hearts. The HS/PSF treatment of the GFP-rMSCs led to a greater fractional shortening (11%) and ejection fraction (8%) compared with the untreated MSCs. Mechanistically, MSCs are known to reduce inflammation and to improve vascularity in ischemic hearts [2,8,43,53], and we believe that the improved survival of the treated MSCs is advantageous.

The transplantation of multipotent cells for the purpose of cardiac tissue repair and possible regeneration has progressed to clinical trials, and enough studies have been conducted to undergo meta-analysis of clinical results [54,55]. However, the duration of survival and engraftment of transplanted cells on the relative effectiveness of repair is poorly understood as most cells do not survive long, with >95% disappearing within the first week. In this study, we demonstrated that in vitro pretreatment of GFP-rMSCs with HS/PSF resulted in improved survival under stress in vitro and greater engraftment and improved heart function in vivo. The viability of transplanted MSCs must be an important component of their effectiveness. In a previous study, MSCs that were preconditioned by 24 h at 1% oxygen increased their glycogen storage, and the expressions of Hif-1α and phospho-Akt, and this promoted their survival in hind limb ischemia models [56]. Similarly, hypoxic preconditioning of human MSCs resulted in increased expression of cMET, the receptor for scatter factor/hepatocyte growth factor (HGF), increased cell motility, and improved hindlimb ischemia recovery [57]. Therefore, the combination of hypoxic preconditioning + HS + PSF may provide yet another boost to MSC survival in in vivo models and therapeutic indications.

Recently, studies have examined the exosomes from cultured MSCs as therapeutic agents [58,59,60]. Exosomes, being non-cellular, may have some perceived advantages over cells for tissue repair/regeneration. However, it may be that continuous production of exosomes from properly engrafted cells provide the greater benefit.

The current study was designed to assess the effects of HS/PSF pretreatment of MSCs on engraftment and heart physiological outcomes. Here, the HS/PSF-treated MSCs demonstrated greater improvement in the physiologic measures than the untreated MSCs in this challenging permanent ligation model. The mechanism for this is most likely related to the well-known secretion by MSCs of anti-inflammatory and angiogenic molecules. The benefit of the treated MSCs may be further improved in future work by (1) titrating the dose of treated MSCs, (2) using a ligation-reperfusion rat model instead of our permanent ligation model, and (3) delivering by iv infusion. We postulate that cellular therapy with other types of stem/progenitor cells could also benefit from improving their early engraftment with similar methods during the first days in vivo.

## 4. Materials and Methods

### 4.1. Cell Culture

All animal procedures were approved by the University of Maryland School of Medicine Institutional Animal care and Use Committee—IACUC protocol #0910004. MSCs were isolated from rat bone marrow as previously described [61] with minor modification. Briefly, the rat bone marrow was harvested from the long bones of male Lewis rats constitutively expressing GFP (strain Lew-Tg(CAG-EGFP)YsRrrc) purchased from the National Rat Research Resource at the University of Missouri. GFP-rMSCs were isolated from bone marrow of femur and tibia bones according to our standard protocol. The bones were cleaned of any tendons or muscle, and bone marrow was extracted by making a needle hole on one end of a bone, cutting off the opposite end, and centrifuging it in a 15 mL tube at 1000 rpm for 5 min. The mononuclear cells were then isolated through density separation using Ficoll density medium (1.077 g/mL, GE Healthcare). The layer of cells at the interphase boundary of Ficoll and serum was collected and diluted with 2 volumes of PBS, and the cells were collected by centrifugation and then plated in complete medium (CM) (DMEM (1 g/L glucose), 10% FBS from MSC qualified lots (Hyclone/GE Healthcare), glutamine, and 1% antibiotic/antimycotic (Life Technologies, Frederick MD)) at 37 °C and 5% CO_2_. The medium was changed at 4 days when single colonies were visible_._ The cells were trypsinized and re-plated at 10–14 days. The cells were then routinely passaged every 3–4 days or when 60–70% confluent and used at passages 3–5 for both in vitro and in vivo experiments. We used GFP-rMSCs to refer to these cells in the text for consistency. GFP-MSCs were prepared from 3 rats at a time on 3 different occasions, cultured to 20 million by passage 3, frozen in complete medium with 10% FCS with 5%DMSO, and stored in liquid nitrogen. No differences were noted from the different preparations.

### 4.2. MSC Labeling with Membrane Dyes and Pro-Survival Treatment

The GFP-rMSCs continued to express the GFP protein in vitro, upon differentiation, and in vivo. However, the GFP signal and the cardiac autofluorescence were often overlapping and difficult to photograph. Instead, in some experiments, the GFP-rMSCs were stained with vital dyes, Hoechst 33,342 (blue nuclei staining), or carboxymethyl-DiI (red membrane staining) (vital dyes were from Life Technologies, Inc., Carlsbad, CA, USA) according to the manufacturer’s instructions 24 h prior to use. For cell treatment with heat shock and the combination of 5 PSFs, the protocol of Laflamme et al. [44] was followed with some minor modifications. In brief, on the day prior to the cardiac injections, cells for the treatment group were heat shocked (HS) for one hour at 42.5 °C and incubated in CM supplemented with IGF-1 (100 ng/mL) and cyclosporine A (200 nM) overnight. On the day of implantation, the cells were incubated for 1 h at 37 °C in CM supplemented with the combination of pro-survival factors including caspase 3 inhibitor II ZVAD (25 nM) (Calbiochem, San Diego, CA, USA), Bcl-xl BH4 (50 nM) (Peprotech, London, UK), insulin-like growth factor-1 (IGF-1) (100 ng/mL) (Peprotech, London, UK), cyclosporine A (200 nM) (Sigma-Aldrich, Burlington, MA, USA), and pinacidil (50 µM) (Sigma-Aldrich, Burlington, MA, USA). The cells were then harvested by trypsin/EDTA, washed with serum-free DMEM, re-suspended in serum-free and phenol red-free DMEM, and stored on ice until injected into the rat hearts, usually within 1–2 h. In all experiments, in vitro and in vivo, treated MSCs refers to HS and PSF (HS/PSF)-treated GFP-rMSCs.

### 4.3. Differentiation Potential of GFP-rMSCs

GFP-rMSCs were tested for their ability to differentiate into adipocytes, osteoblasts, and chondrocytes as described previously [61]. Briefly, for adipogenic differentiation, GFP-rMSCs were cultured in CM to achieve confluency. Then, the CM was replaced with adipogenesis medium containing 10 µg/mL insulin, 1 µM dexamethasone, 0.5 mM isobutyl methylxanthine, 0.2 mM indomethacin, 10% fetal bovine serum (FBS), and 5% rabbit serum for 3 days, followed by 24 h in maintenance medium (CM + 10 µg/mL insulin and FBS), and this was repeated for 3 cycles prior to analysis. Adipocytes were analyzed by Nile red and Oil-Red-O staining. For osteogenic differentiation, 5 × 10^3^ cells/cm^2^ were seeded into 6-well plates. Osteogenic differentiation was induced in DMEM with 10% FBS, 1% antibiotic-antimycotic, 50 µM ascorbate-2-phosphate, 10 mM β-glycerol phosphate, and 100 nM dexamethasone for 14 days. Differentiation was evaluated by alkaline phosphatase staining and Von Kossa staining. Chondrogenic differentiation was achieved by micromass culture of the GFP-rMSCs, where 250,000 cells were centrifuged to the bottom of polypropylene tubes and incubated in 250 µL serum-free DMEM (high glucose 4.5 g/L) containing 10 ng of TGFβ. The medium was changed every other day for 3 weeks, and histological sections were cut at 5 µm and stained for proteoglycans with safranin O. Differentiation experiments were performed in duplicate.

### 4.4. Hydrogen Peroxide Stress Assay

For challenge with hydrogen peroxide, GFP-rMSCs were cultured in CM to reach ~70% confluence. Fresh hydrogen peroxide was added to CM at indicated concentrations immediately prior to use and exchanged with the CM on the GFP-rMSCs. Cells were incubated in hydrogen peroxide-containing medium (HM) for up to 4 h at 37 °C prior to analysis by flow cytometry and lactate dehydrogenase assays. The effect of hydrogen peroxide treatment was also tested on the in vitro replating/reattachment of GFP-rMSCs. Stress assays and recovery were performed three times.

### 4.5. Flow Cytometry

The GFP-rMSCs in each culture well are collected in separate tubes, washed with PBS, and stained with anti-annexin V antibody and 7-AAD for measurement of cell injury/death. Staining was performed for 45 minutes at room temperature in the dark, followed by three PBS washes. Cell staining was analyzed using a LSR II Flow Cytometer (BD Biosciences, San Jose, CA, USA) immediately following staining. All annexin V-positive cells were counted as dying cells, and the percentage of annexin V-positive cells out of the total MSC population was recorded in each treatment group.

### 4.6. Lactate Dehydrogenase (LDH) Viability Assay

GFP-rMSCs were cultured and hydrogen peroxide treated in 96-well plates. The culture media from the wells were used in an LDH assay to measure cytotoxicity caused by hydrogen peroxide in each well. The assay was performed in triplicate using an LDH-Cytotoxicity Colorimetric Assay Kit II (BioVision, Inc., Milpitas, CA, USA) according to the manufacturer’s instructions with some minor modifications. The plates were centrifuged at 1500 rpm, and a medium was used in the assay. Higher levels of LDH indicated more cell death in the wells. The values were normalized to those of wells with media and no cells.

### 4.7. Apoptosis Protein Array

Apoptosis protein arrays (R & D Systems Inc., Minneapolis, MN, USA) were used to analyze expression of apoptotic proteins in cells before and after stress assays. Protein samples were prepared and labeled according to manufacturer’s instructions. Briefly, membranes were blocked and then incubated with protein samples overnight. The next day they were incubated with detection antibody cocktail and Streptavidin-HRP, per instructions. Protein expression was detected by chemiluminescence on X-ray films, and the intensity of the dots representing each protein was compared (data not shown). Array data was used to identify altered expression of proteins including heat shock protein 70 (HSP 70), hypoxia inducible factor-1 alpha (Hif-1a), and superoxide dismutase 1 (SOD1).

### 4.8. Western Blot Methods

Total cell lysates were prepared in radio-immune precipitation assay (RIPA) buffer (Pierce Chemical Inc., Rockford, IL, USA) containing a cocktail of protease inhibitors (10 μg/mL aprotinin, 10 μg/mL leupeptin, and 10 μg/mL pepstatin, all from Sigma-Aldrich Chemical, St. Louis, MO, USA). Western blotting was performed according to standard protocols. In brief, protein samples were separated on 4–12% gradient gels and transferred using an iBlot transfer system (Life Technologies, Carlsbad, CA, USA), according to the manufacturer’s instructions. Membranes were blocked for non-specific binding in 5% non-fat dry milk and 1% goat serum TBS+Tween (TBST) buffer for 1 h at RT, incubated with primary antibodies for 1 h at RT and with HRP-conjugated secondary antibodies for 45 min at RT. The antibodies used for Western blotting were from R&D Systems, Inc., Minneapolis, MN, USA and included Hif-1A (monoclonal MAB1536, clone 241809), HSP 70 (monoclonal HSPA1A, clone 242707), SOD1 (polyclonal goat IgG AF3418), and GFP (polyclonal goat IgG AF4240). The membranes were washed in TBST, and antibody labeling of heat shock protein 70 (HSP 70), hypoxia inducible factor-1 alpha (Hif-1a), superoxide dismutase 1 (SOD1), and GFP was detected by chemiluminescence on X-ray films (Kodak, Rochester, NY, USA).

### 4.9. Infarct Studies

All animal procedures were approved by the University of Maryland School of Medicine Institutional Animal care and Use Committee—protocol #0910004. Ligation of the Left Anterior Coronary Artery and Delivery of Untreated or HS/PSF-treated GFP-rMSCs. Recipient adult female Lewis rats (~250 g) were purchased from Harlan Laboratories (Frederick, MD, USA). As previously described [62], the rats were anesthetized with isoflurane (induced with 3–4% and maintained with 2–2.5% in pure oxygen) and ventilated mechanically through oral intubation (15 mL/kg for 50–60 cycles/min). Through a left thoracotomy at the 3rd–4th intercostal spaces, the chest and the pericardium were opened, and the left anterior descending (LAD) coronary artery was permanently ligated using a 7–0 prolene suture. Following the ligation, 2 million untreated or HS/PSF-treated GFP-rMSCs in phenol red-free DMEM were injected via a modified 28 g needle in a total volume of 200 uL for four injection sites around the ischemia region. The vehicle control rats received injection of phenol red-free DMEM. The wound was closed in layers and buprenorphine was administered for pain relief (0.03 mg s.c. every 12 h for 24–72 h). Animals for sham surgery received thoracotomy, loose placement of prolene suture around the coronary artery without ligation and injections of phenol red-free DMEM. Surgeries were planned to infarct and treat 3–4 rats in a day, generally 1 rat in each group or 2 rats in a group as needed to fill in the treatment matrix. In some experiments, the infarcted hearts received a mix of 1.0 × 10^6^ HS/PSF-treated GFP-rMSCs labeled with the membrane dye Di I and 1.0 × 10^6^ untreated GFP-rMSCs labeled with the DNA dye Hoechst 33,342 (2.0 × 10^6^ MSCs per heart) in the 4 × 50 µL injections. These dyes did not affect the in vitro growth, differentiation, or viability of the cells.

### 4.10. Echocardiography

Rats were anesthetized with isoflurane inhalation via a nose cone (3–4% in pure oxygen for induction and 1.2–1.5% for maintenance). As described previously [63], the B- and M-mode images of the left ventricle were obtained via parasternal long and short axis views using a high-frequency ultrasound system (Vevo 2100, Visual Sonics, Toronto, ON, Canada). Data were calculated as previously described [63] with the formulae that have been validated in rats [64].

### 4.11. Statistics

In studies reported here, the statistical analysis was performed by the Student *t*-test with Bonferroni correction, in which a treatment group and a control group, or two treatment groups were compared as unpaired sets. In all experiments, *p* < 0.05 was considered significant. In vitro studies were performed 3–4 times for reproducibility of data.

## Figures and Tables

**Table 1:** Echocardiographic measurements demonstrated that HS/PSF-treated MSCs were more effective than untreated MSCs for improving recovery after infarction.

	Sham	MI + Vehicle	MI + MSCs	MI + HS/PSF MSCs
n=	7	7	7	8
BW (g)	337 ± 64	353 ± 36	335 ± 117	351 ± 110
HR (bpm)	367 ± 47	367 ± 28	374 ± 44	366 ± 34
LVDd (mm)	6.11 ± 0.83	7.65 ± 0.55 **	6.87 ± 0.81 *	7.34 ± 0.76 **
LVDs (mm)	3.11 ± 0.47	5.70 ± 0.77 **	4.56 ± 0.56 **##	4.57 ± 0.50 **##
LVPWd (mm)	1.06 ± 0.27	1.43 ± 0.25 *	1.25 ± 0.31	1.31 ± 0.36
LVPWs (mm)	1.70 ± 0.33	2.09 ± 0.21 *	1.84 ± 0.50	1.89 ± 0.39
LVEF (%)	79.4 ± 3.4	48.6 ± 10.5 **	61.0 ± 4.3 **#	66.3 ± 2.6 **##@
LVFS (%)	49.0 ± 3.9	25.7 ± 6.7 **	33.7 ± 3.2 **#	37.8 ± 2.0 **##@
SV (µL)	152.2 ± 3.5	150.6 ± 32.7	152.1 ± 42.5	190.8 ± 43.5
SV/BW (µL/g)	0.46 ± 0.15	0.43 ± 0.10	0.52 ± 0.21	0.65 ± 0.20 #
CO (mL/min)	54.8 ± 14.1	55.1 ± 11.3	55.5 ± 10.1	69.3 ± 14.5
CO/BW (mL/min/g)	0.17 ± 0.06	0.16 ± 0.03	0.19 ± 0.07	0.24 ± 0.07 #
LV mass (mg)	265.3 ± 79.4	552.4 ± 108.6 **	431.0 ± 156.4 *	466.2 ± 146.3 **
LVmass/BW (mg/g)	0.79 ± 0.23	1.47 ± 0.20 **	1.43 ± 0.66 *	1.51 ± 0.23 **

Abbreviations: BW, body weight; HR, heart rate; LVDd and LVDs, left ventricular dimension at diastole or systole; LVPWd and LVPWs, posterior wall thickness at diastole or systole; LVEF, ejection fraction; LVFS, fractional shortening of LV dimension; SV, stroke volume; CO, cardiac output. *, ** *p* < 0.05 or 0.01 vs. sham. #, ## *p* < 0.05 or 0.01 vs. MI ++ vehicle. @ *p* < 0.05 MI + MSCs compared to MI + HS/PSF MSCs.

**Table 2:** Pro-Survival Factors (PSF) used in this study.

Insulin-like Growth Factor 1 (IGF-1, 100 ng/mL Peprotech, London, UK)
Cyclosporine A (200 nM, Wako Pure Chemicals, Tokyo, Japan)
Pinacidil (50 mM, Sigma, Burlington, MA, USA)
Bcl-XL BH4 (cell-permeant TAT peptide, 50 nM from Calbiochem, San Diego, CA, USA)
Caspase 3 Inhibitor II ZVAD (100 mM, Calbiochem, San Diego, CA, USA)
Non-PSF medium was DMEM alone (phenol red free)

## Data Availability

Please contact the authors if further data desired.
